# Mixed integer programming model for optimizing the layout of an ICU vehicle

**DOI:** 10.1186/1472-6963-9-224

**Published:** 2009-12-08

**Authors:** Javier Sánchez Alejo, Modoaldo Garrido Martín, Miguel Ortega-Mier, Álvaro García-Sánchez

**Affiliations:** 1 Department of 'Ingeniería de Organización, Administración de Empresas y Estadística', Escuela Técnica Superior de Ingenieros Industriales, Universidad Politécnica de Madrid, (José Gutiérrez Abascal 2), Madrid, (28002), Spain; 2 Hospital La Fuenfría, SERMAS, Carretera de las Dehesas s/n, Cercedilla-Madrid, (28479), Spain

## Abstract

**Background:**

This paper presents a Mixed Integer Programming (MIP) model for designing the layout of the Intensive Care Units' (ICUs) patient care space. In particular, this MIP model was developed for optimizing the layout for materials to be used in interventions. This work was developed within the framework of a joint project between the Madrid Technical Unverstity and the Medical Emergency Services of the Madrid Regional Government (SUMMA 112).

**Methods:**

The first task was to identify the relevant information to define the characteristics of the new vehicles and, in particular, to obtain a satisfactory interior layout to locate all the necessary materials. This information was gathered from health workers related to ICUs. With that information an optimization model was developed in order to obtain a solution. From the MIP model, a first solution was obtained, consisting of a grid to locate the different materials needed for the ICUs. The outcome from the MIP model was discussed with health workers to tune the solution, and after slightly altering that solution to meet some requirements that had not been included in the mathematical model, the eventual solution was approved by the persons responsible for specifying the characteristics of the new vehicles. According to the opinion stated by the SUMMA 112's medical group responsible for improving the ambulances (the so-called "coaching group"), the outcome was highly satisfactory. Indeed, the final design served as a basis to draw up the requirements of a public tender.

**Results:**

As a result from solving the Optimization model, a grid was obtained to locate the different necessary materials for the ICUs. This grid had to be slightly altered to meet some requirements that had not been included in the mathematical model. The results were discussed with the persons responsible for specifying the characteristics of the new vehicles.

**Conclusion:**

The outcome was highly satisfactory. Indeed, the final design served as a basis to draw up the requirements of a public tender. The authors advocate this approach to address similar problems within the field of Health Services to improve the efficiency and the effectiveness of the processes involved. Problems such as those in operation rooms or emergency rooms, where the availability of a large amount of material is critical are eligible to be dealt with in a simmilar manner.

## Background

### Introduction

Health systems rely highly on their emergency subsystems and, in particular, on those vehicles that allow them to attend patients outside health facilities. This paper presents the main results of a project that was intended to improve the design of the interior patient care space of Intensive Care Units (ICUs) of the Medical Emergency Services in the Madrid Region (SUMMA 112). ICUs are vehicles which provide advanced life support for health emergencies. This project was conducted by the Madrid Technical University (Universidad Politécnica de Madrid, hereafter UPM).

Other previous works have focused on problems regarding the design of ambulances. From quite early on, research has been concerned with this sort of problems. This is the case of the National Academy of Engineering (1970) which addressed the issues related to designing ambulance interiors in terms of ergonomic factors [1]. The topic has not lost any interest, and more recent works can be found as well, such as that of Ferreira (2005) [2]. What we present in this paper shares the focus of these studies on ambulance design, and more particularly, it addresses the problem of locating materials in the ambulance interior to facilitate heath workers' tasks and, thus, improve both the efficiency and the effectiveness of their interventions.

### The SUMMA

The paper is structured as follows. In section 1, the framework in which the work was developed is presented. Section 2 contains some general remarks regarding the design of ICUs. In section 3, the objectives and the methodology adopted to obtain a design are presented. Following, in section 4, the main design factors taken into account for this work are described. The optimization model is presented in section 5 and the solution obtained from it as well as the final design are presented in section 6. Some final conclusions are offered in section 7.

Among other services, SUMMA 112 offers health assistance in emergencies that occur within the Madrid Region, covering 8,028 square kilometres with a population that numbers 6 million. In fact, for emergency interventions (those where the patient's life is in) three assistance mechanisms are available and deployed throughout the Madrid Region namely, Rapid Intervention Vehicles (RIV), Sanitary Helicopters (SH), and the above mentioned Intensive Care Units (ICUs).

The SUMMA 112 subsystem, the object of this study, manages its regular activity from a radiotelephone communication centre, known as the Emergency Coordination Service (ECS). The ECS is the SUMMA 112 call centre, and receives, classifies and directs the incoming calls from the helpline numbers (112 and 061). Besides, the ICUs, which are ultimately responsible for attending emergencies, receive their assignments from the ECS. The ECS is the core of the medical response system for catastrophes or multiple victim accidents. This way, the ECS manages and is responsible for providing (with its UCIs) the medical transportation for patients within that service and which may have previously been attended by other Madrid Region resources, namely, Primary Health Centres (Primary Healthcare) and Hospitals (Specialised Healthcare).

The idea of a unified medical emergency service materialized in 2002, when the Madrid Regional Government took over medical competences transferred from the national government. Prior to that, there were two different services for attending medical emergencies:

• The National Emergency Service 061, which depended on INSALUD, founded in 1989 as an emergency coordination centre with a unified telephone number, and

• The Madrid Regional Government Rescue and Emergency Service, SERCAM, founded in 1998, which used to depend on firefighters, and was later transferred to the Health Ministry within the Madrid Regional Government.

In April 2003, SUMMA 112 was created as a service in charge, by law, of Madrid's medical emergency management, external emergencies, 061s, ICUs and the Madrid Regional Government Rescue and Emergency Service (SERCAM).

Prior to the work presented in this paper, SUMMA 112 had 23 ICUs for health emergencies. These ICUs used to belong to two different original service fleets: SERCAM and INSALUD 061. That is the reason why the mobile pool is so heterogeneous, leading to lots of problems both related to management and maintenance tasks. As a result, many complaints have been received from health workers since quite often they have to work in non-standard units, which greatly hinder their paramedic activities. Besides, SUMMA 112 is near to becoming overloaded due to an obsolete fleet and a high number of non-operative units as a result of mechanical problems.

To handle any existing problems and to attend complaints about the equipment, SUMMA 112 appointed a commission, whose task was to identify the ICUs' main necessary improvements. This team, named "coaching team", was intended to use a multi-disciplinary approach. For that purpose, almost every collective concerned with the service of these vehicles was represented. Therefore, the group contained doctors, nurses, hospital porters, transport technicians, drivers, pharmacists, etc.

Once the need to renew and standardize ICUs was identified and the appropriate amount of resources was committed to that purpose, a team of researchers from Madrid Technical University was asked to coordinate and to lead the process of designing a new ICU. The objective was to design a new ICU that would meet several demands. First, workers' contributions should be taken into account; new equipment should be suitably fitted; in addition, the assistance procedure should continue as normal; and, finally, ergonomics, safety, health and hygiene should be improved.

The conclusions of the project served as the basis for the specifications sheet for a public tender for medical equipment renewal. This renewal included the acquisition of 32 new ICUs which would be in service 24 hours a day, 365 days a year, with the support of 8 backup units, which could become active if any other one in service failed. The selection of the attendance vehicle was also a matter of study for the UPM, although such a work is beyond the scope of this paper.

The inputs needed to carry out the work were the following ones:

• The current designs of ICU patient care spaces.

• The list of the main deficiencies detected by health specialists.

• The list of the emergency interventions attended during the previous year by ambulance, classified and ranked according to their frequency and seriousness.

• For every kind of clinical task, according to the SUMMA 112 action protocol, a description of the position and the actions to be performed by each of the health specialists (namely, the doctor, the nurse and the transportation technician), as well as the equipment and materials each one needs.

• The applicable standard legislation, especially Annex 2 of the Spanish R.D. 619/1998 of April 17, and the Standard UNE-EN 1789, "Health transportation vehicles and their equipment. Road ambulances".

• The new design requirements demanded by the SUMMA 112 managers for the new public tender (maximum cost, amount of on-board medicaments, average number of interventions without medical material replenishing, etc.)

This paper does not present the whole project, but focuses on that part related to the design of the patient care space, in particular, the location of the materials and medical-sanitary equipment inside it. For that purpose an optimization model was formulated and exploited.

Health transportation consists in moving persons in specially equipped vehicles to attend them because of their being ill, injured or in need of assistance for one health reason or another. Depending on their function, ambulances can be classified into either non-patient care ones (that can only take a patient on a stretcher) or patient care ones (that can take qualified medical personnel on board). In its turn, patient care ambulances are also divided into two types: those without medical personnel (intended only to offer basic life support) and those with medical personnel or fully equipped with all necessary material (able to offer advanced life support).

These last ones are referred to as mobile ICUs, and have a crew that consists of a doctor, a nurse and a health transportation technician (who remain in the vehicle interior attending the patient), as well as a driver in the cab. These vehicles are equipped with a huge number of health devices and medical -pharmaceutical material, which enable all kinds of assistance to be provided to the patient until the ICU arrives at the hospital.

### The problem

The ICU design process is highly complex, especially the task of selecting and locating all the medical and health materials. The reason for this complexity lies in the fact that a particular piece of equipment will be used by different health workers depending on the kind of intervention. The SUMMA 112's clinical task protocol states who will use each device or piece of material for every possible intervention. So, since every health worker is in a fixed position relative to the patient on the journey, it is quite frequent that workers' arms and hands interfere with each other while performing some actions because they need to simultaneously reach materials far from themselves and close to someone else. As a matter of fact, the most frequent and serious complaint arising from the bodywork group meetings in relation to the old ICUs was that "furniture layout makes it difficult to carry out some interventions due to the disorder and difficulty in reaching some material". In the same sense, it was said that "the location of some necessary elements is sometimes very far from the person who needs them".

In order to produce an ambulance, a standard van (which, for example, may be used to deliver goods) serves as a basis. This standard van is then appropriately altered and turned into an ICU. The choice of the base model depends on the engine size, the interior space (around 12 *m*^3^, which becomes quite reduced before being altered), as well as other characteristics such as the ease with which it can be altered.

The requirements that any SUMMA 112 ICU must meet are stated in Annex 2 of the Standard UNE-EN 1789, "Health transportation vehicles and their equipment. Road ambulances". An ICU's engine power is usually somewhere around 140 HP. These vehicles are 5.6 m long, 2.1 m wide and 2.5 m high and their net load is around 1,500 Kg. They do not always have pneumatic suspension, which results in less comfort both for the patient and for the health personnel who must suffer a high level of vibrations all day long. As well as a big double back door, they must be equipped with a side door wide enough to allow the health specialists in and out of the ICU.

Currently, vans from the main global car makers are the most frequently used, namely: Mercedes Sprinter, Volkswagen LT, Citroen Jumper, Peugeot Boxer, Renault Master, Fiat Ducato, Opel Movano, Ford Transit and Iveco Daily.

Once the model has been selected, a car-body maker performs the necessary alterations, as well as the installation of all the equipment and the internal furniture, this process being as costly as the off-the-shelf vehicle itself.

The vehicle's car-body making is, therefore, the most important process the vehicle undergoes. The car-body maker receives a standard van and delivers a ready-to-use ICU. A correct alteration, together with an appropriate layout and the necessary advanced equipment, will enable the ICU to meet the high number of requirements which will be demanded of it during its life time.

The complete alteration from a van into ICU entails several aspects that must be addressed separately as well as a whole. These aspects are the following ones:

1. Habitability or initial alteration. Off-the-shelf vans need first to be altered, mainly by removing elements to obtain the base design to which to add materials, equipment and systems.

2. Electrical installation, since the ICU equipment will require a high amount of reliable energy supply.

3. Internal lighting, which is of paramount importance for the correct performance of medical - health personnel.

4. Materials and equipment, selected according to operating protocols that must be carefully analysed and calculated.

5. Vehicle signalling, so that it allows other drivers and pedestrians to quickly recognise the ICU and therefore, to allow ICUs to safely transit public roads and streets.

6. Air conditioning, which entails installing a separate system for the patient care space, different from the original one within the driving cab.

7. Oxygen therapy, consisting of two circuits, a main one and an auxiliary one, which would work if the first one fails. Finally, in order for the ICU to get permission to be driven, the vehicle must obtain an approval, since it has undergone structural, mechanical and electrical changes, or as it is called, "major alterations". The ICU, thus, is quite different from the van originally delivered by the car maker and needs to be suitable to circulate.

## Methods

### Introduction

Operational Research (OR) has been used for a wide range of problems. More specifically, some decision making processes related to ambulances have been addressed by means of OR models. Some of them have been used for vehicle design purposes, but others have helped design an operating strategy for offering a certain service level within a region, such as in Ibraheem Alsalloum (2006) [3], Takeda et al (2007) [4] and Araz (2007) [5].

The ambulance interior design problem shares some features with that of obtaining a layout in industrial facilities. Indeed, this problem consists, roughly, in locating different pieces of equipment so that the processes involved can be performed effectively and efficiently. OR has also been used for this problem as well for quite a long time, and still is. A comprehensive study of the application of OR to this sort of problem can be found in Meyers (1995) [6]. Other examples that can be found are Kelachankuttu (2006) [7] and McKendall (2007) [8], which are fairly recent examples of this.

Heuristic and meataheuristics have also been adopted to address layout problems, as in Bland (1991) [9], where the authors use a Tabu Search approach for designing circuit designs. There have been other similar works, among which and more recently, in Lou (2008) [10] a Tabu Search is used for a layout problem outperforming existing heuristics.

The main objective to be accomplished was to obtain a satisfactory layout for the patient care space of the ICUs. That meant that all the materials and equipment should be available and properly located so that the most critical interventions could take the minimum possible amount of time and be carried out in a quite comfortable way, avoiding the interference of health personnel as much as possible. For that purpose, materials ought to be close enough to whoever needed them. Besides, the layout must enable the most frequent interventions to be performed with equal levels of swiftness and comfort. Aditionally, the layout must meet the following requirements which are thus objectives to be accomplished as well. Firstly, health personnel should not suffer any difficult, uncomfortable or risky positions and movements. Secondly, the patient conditions should be suitable. And finally, the technical and dimensional features of the vehicle should be properly used.

**Figure 1:**
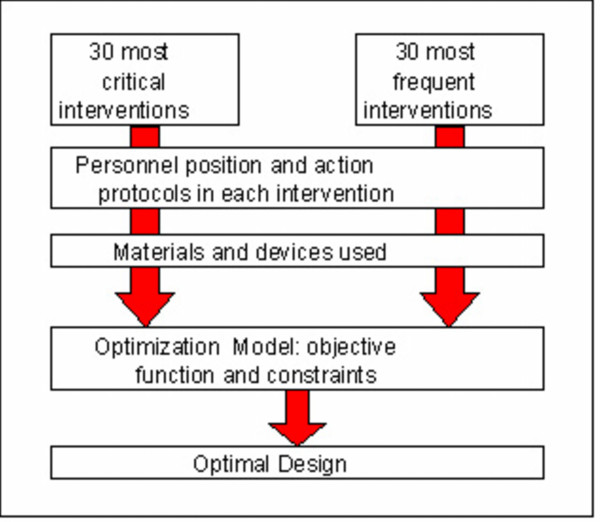
**Research methodology**. Figure 1 shows how the project was conducted from the identification of the most important interventions to the eventual design

The methodology followed to accomplish those objectives, Figure 1, consisted in analyzing the different factors that condition the position of materials and devices, and the constraints to be satisfied. With this information an optimization model was formulated, where:

• Each collection of variables values represents a possible solution, that is, an arrangement of materials inside the vehicle patient care space.

• A collection of constraints represent the sort of requirements a particular arrangement must meet so that it is feasible and, therefore, eligible to be the optimal solution.

• Finally, the objective function, which allows assessing the suitability of a solution and guides the process towards the optimal solution.

The techniques and tools developed for solving optimization models are widely known. Currently, different solvers are available in the market. In particular, the model developed for this project was solved with a well-known widespread software package.

The more important data to be taken into account are the following:

• Medical-health material characteristics.

• Medical-health equipment characteristics.

• Analysis and frequency of the 30 most critical interventions during the previous year.

• Analysis and frequency of the 30 most frequent interventions during the previous year.

• Health personnel position and intervention protocol for each of the previous processes.

• Materials and equipment used by each worker in each of the previous processes.

Since in order to build an optimization model some assumptions were made. After obtaining the optimal solution for this model, the solution had to be discussed and properly adapted. Therefore, all main considerations were modelled, whereas some minor matters, consisting of a collection of restraints demanded by the bodywork group and some imposed by the actual van features were left out of the model and considered when obtaining a final layout for the ICUs from the optimal solution obtained from the optimization model.

As previously stated, several factors were analysed in order to properly formulate the model. All the necessary information was provided by the medical staff from SUMMA 112. The researchers from the UPM carefully selected a suitable level of detail so that the model was both valid and simple enough for the purpose of the study.

### Medical-health material characteristics

ICUs are equipped with a great number of medical-health materials that are used when carrying out clinical tasks, ranging from gloves and splints to drugs and disposable syringes. Indeed, medical, pharmacological, perishable, immobilization materials, etc, should be available in case of need when attending patients within the range of emergencies the ICUs are devised for. In Spain, the above mentioned Annex 2 of the Spanish R. D. 619/1998 of April 17 establishes the list of different materials and the quantities to be found in ICUs, though SUMMA 112 set the policy of including not only the amounts according to the law but, in addition, all the necessary material needed to be able to attend any emergency previously recorded ever since the service was founded.

Not only is it important that all material is available, but having the right amounts of it to be able to attend the number of cases which may occur during a shift is also of paramount importance. SUMMA 112's ICUs operate without any need to return to any facilities for replenishment after every intervention.

That is the reason why ICUs should be equipped for the worst case scenario, where several interventions need the same material. Since the average number of interventions during a shift is five, the amount of each material is five times the amount required for the most demanding intervention. Besides, as a safety measure, the total amount was increased a 10%.

In total, 343 different materials are used. These were sorted into twelve different categories: pharmacology, fluid therapy, antiseptics, oxygen therapy, cardiovascular, air-conduction, probing and drainage, surgery, orthopaedic surgery, gastric lavage, cloths, perishable material and others. In order to formulate the model, the appropriate data had to be gathered, namely, the necessary amount of each material, the person who requires each material when doing clinical tasks and whether each material is necessary or not in the top 30 most frequent of the top 100 most critical interventions.

### Characteristics of the medical practitioner equipment

Vehicles intended to provide urgent assistance to people who have suffered an accident or who are in a critical state must have the appropriate equipment to guarantee rapid attention with the required quality in extreme situations. As is the case with medical material, Annex 2 of the Spanish R. D. 619/1998 of April 17 establishes the minimum equipment for this sort of vehicle [11].

The main pieces of equipment are a suction unit, some flow metering devices, an electrocardiograph, a pulsioximeter, two defibrillators (a portable one and a fixed one), two infusion pumps (volumetric and syringe type), three respirators and a mobile incubator. Firstly, specific brands were selected among the different available options for each piece of equipment, and a descriptive leaflet was made, where the main features were listed. This leaflet included the following specifications regarding the material located within the vehicle's interior:

• who needs each piece of equipment for the top 30 most frequent of the top 100 most critical interventions;

• Equipment dimensions, for its positioning;

• Technical specifications;

• Whether only one or several persons may need a particular piece of equipment in different interventions, in order to avoid interference when accessing it;

• Safety measures to take into account.

All this information was necessary to build an appropriate model to accomplish the interior design process.

### Top 30 most critical interventions

In critical interventions, personnel movements need to be precise and fast, materials should be available when required and interferences can cause great trouble, since the patient may not receive proper care in time. In order to obtain a layout which could satisfy those requirements, the top 30 most critical interventions were listed and characterised with the following data (see Table 1):

**Table 1:** Some of the most critical interventions according to the SUMMA 112 protocol terminology.

Diagnosis	Intervention
Endocrine, nutritional, metabolic and immunity	Hyperosmotic coma
	Hypoglycaemic coma
Mental disorder	Delirium
	Cocaine poisoning
	Opioid poisoning
	Frequent pharmacological poisoning
Circulatory system	Acute myocardial infarction
	Pulmonary thromboembolism
	Cardiorespiratory arrest
	Subarachnoidal haemorrhaging
	Aortic aneurysm
Respiratory system	Acute respiratory infections
	Acute bronchitis
	Respiratory tract obstruction
	Pneumothorax Pressure
Pregnancy, delivery and puerperium Ill-defined symptoms and states	Eclampsia
	Cardiogenic shock
	Hypovolaemic/septic shock
Injuries and poisoning	Closed fracture
	Open fracture
	Multiple trauma/traumatic shock
	Amputations
	Burns
	Carbon monoxide poisoning
	Hypothermia
	Heat disorder
	Anaphylactic shock
	Electrocution/electric flashes
External causes	Explosion and bomb trauma
	Knife or firearm wound

Table 1 shows some of the most critical interventions and grouped according to the diagnosis.

1. definition,

2. clinical picture,

3. emergency treatment,

4. necessary material and equipment,

5. frequency of occurrence and

6. location and movement of health personnel

With these features, experienced members were required to assign a level of criticality for each intevention, to have some quantitative estimate of the relative importance of every intervention.

### Top 30 most frequent interventions

Although critical interventions are of great importance, there are others which occur very frequently and should receive proper attention as well, since they account for a great amount of the intervention time. As with the most critical interventions, the most frequent ones were listed and characterized (see Table 2): definition, clinical picture, emergency treatment, necessary material and equipment, frequency and location and movements of health personnel.

**Table 2:** The thirty most frequent interventions and the corresponding number of ambulance's outing over a year.

Description	Frequency
Syncopal attack/blackout	1880
Stable - Unstable Angina	1772
Compulsive disorder	1494
Panic/Anxiety disorder	1409
Chest pain	1092
Hypoglycaemia	862
Bruising and injuries	802
Attempted suicide	592
Myocardial infarction	526
Respiratory infection/bronchitis	494
Auricular fibrillation	403
Alcoholic intoxication	399
Febrile syndrome/fever	369
Bowel haemorrhaging	339
Acute pulmonary oedema	291
Benzodiazepine intoxication	258
Hypertensive disorder	247
Tachycardia	232
Stroke	229
Bradyarrhythmia	219
Abdominal pain	216
Respiratory insufficiency	191
Death with CPR	172
Dyspnea	154
Acute asthma	142
Ischaemic cardiopathy	140
Unspecific dizziness	138
Cardiorespiratory arrest	119
Allergic reaction/urticaria	114
Hypotension	109

Table 1 shows some of the most frequent interventions sorted by their decreasing frequency value.

The criteria for selecting these interventions was the risk of death or serious injury if not quickly and adequately attended. With these features, the "coaching team" was required to assign a level of criticality for each intervention, to have some quantitative estimate of the relative importance of every one.

Therefore, these interventions were given a criticality index, so that both the most critical and the most frequent interventions could be compared and receive the right relative importance when building the model and, thus, when designing the layout.

### Operating protocol

Afterwards, those main interventions had to be carefully analysed to obtain a suitable set of data regarding how interventions are performed.

### Materials and equipment that workers use for every intervention

Other input data of the model were the pieces of equipment that every member of the team needed for every intervention.

### Optimization model

Following, the model is presented. For that purpose, the variables and parameters are listed first. Then, the model itself is formulated. Afterwards, the result is commented and the final solution is presented according to the model and the remaining inputs to be taken into account.

The problem consists in defining the layout of the larger interior wall of the vehicle (in Spain on the right of the vehicle), and assigning where every piece of material should be placed.

In particular, the wall can be considered to be rectangular, and subdivided into a number of square cells (each wide and high), so that the wall is *I *cells wide and *J *cells high. This wall must contain *M *different materials. Materials pose shape constraints so that a minimum number of horizontal cells (*R*_*m*_) and a minimum number of vertical cells (*H*_*m*_) are needed to locate an item of material m. For the sake of simplicity, only rectangular shapes are accepted.

**Figure 2:**
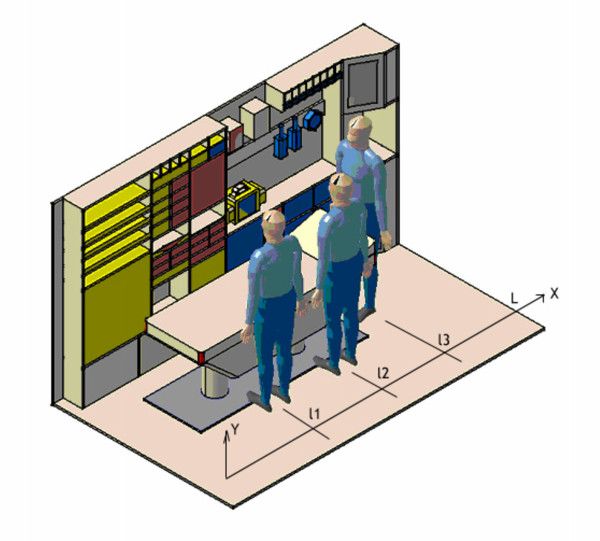
**Personnel location when intervening**. Figure 2 shows how the health workers are located in relation to the patient and the material while intervening.

Besides, due to handling issues, some materials should not be under or below a certain level. Personnel in the vehicle are located as in figure 2, in terms of the *x*-axis health workers' locations are *l*1, *l*2 and *l*3. Finally, the objective function to minimize computes the weighted distance of each piece of equipment to the different workers, in order to avoid as many interferences as possible.

### Parameters

These are the parameters necessary to build the model.

*I*: Total number of rows

*J*: Total number of columns

*P *: Total number of interventions

*M *: Total number of different materials

*S*: Number of heath workers

Δ_*x*_: Cell width

Δ_*y*_: Cell height

*R*_*m*_: Minimum number of cells that are needed to fulfil the requirements of material *m*.

: Height above which material *m *cannot be placed

: Height bellow which material *m *cannot be placed

*MH*_*m*_: Number of horizontal cells required to place material *m*

*MV*_*m*_: Number of vertical cells required to place material *m*

*α*_*smp*_: 0, if worker *s *does not need material *m *in intervention *p*; 1, otherwise

*W*_*p*_: Relevance index, for intervention *p*, which depends on its frequency and its relative importance

: Left most column within reach of worker *s*

: Right most column within reach of worker *s*

### Variables definition

The action variables of the problem, according to which materials will be located are the following ones *x*_*ijm *_= 1 if the cell situated in the *i*-th position along the *x*-axis and the *j*-th along the *y*-axis contains material *m*

*x*_*ijm *_= 0 if the cell situated in the *i*-th position along the *x*-axis and the *j*-th along the *y*-axis does not contain material *m*

Additionally, some other variables are needed for the sake of building the model: : binary variable.

*i *= 1... *I j *= 1... *J l *= max{1, *j *- *H*^*m *^+ 1, ..., *j*} *m *= 1... *M*

: binary variable. *i *= 1... *I j *= 1... *J l *= max{1, *j *- *H*^*m *^+ 1, ..., *j*} *m *= 1... *M*

These variables are used to make sure that allocation of cells do not violate shape constraints, both vertically and horizontally. This will be explained later.

### Objective function

The objective function is an index of the weighted distance from workers to their materials for the most frequent and critical interventions. This is the function to minimise.(1)

The objective function consists of three terms, where each term refers to a worker. For example, the first term (corresponding to worker 1) is a sum over all interventions (*p *= 1... *P*). The contribution for each intervention depends on its weight (*W*_*p*_), the materials involved in that intervention and their location and on whether worker 1 needs each material or not. Therefore, if for a particular intervention *p *material *m *is not needed (*α*_1*mp *_= 0), this will not contribute to the objective function, otherwise it will (*α*_1*mp *_= 1), and its contribution will be proportional to the distance from the location of worker 1 to the cell where the material *m *is located, where this distance can be computed by *i *· Δ*x*. The terms for workers 2 and 3 are built likewise. The distances from these workers to different materials have different expressions according to the defined coordinates.

### Constraints

#### Unicity of allocation

Every cell can contain one and only one type of material, the grill is thin enough so that two materials cannot be allocated to the same cell.(2)

For every particular cell, defined by the values of *i *and *j*, only a single variable *x*_*ijm *_can equal 1, if any at all. By imposing this constraint only a single type of material can be assigned to a particular cell.

#### Minimum material requirements

There should be a minimum amount of cells assigned for every type of material.(3)

There are as many constraints of this type as materials. The first term of the inequality represents the total amount of cells which contain material *m*. The second term refers to the minimum required quantity of cells with material *m*. The total amount available for any possible layout should provide at least the mimimum quantity needed for every material, thus the inequality sign.

### Shape constraints

#### Horizontal constraints

To explain how these constraints work, let us first suppose fixed values for *m*, *i *and *j*. For every three possible values of these indices. There are several variables  with *l *= max{1, *j - M H*^*m *^+ 1}. Of all these variables, according to the second set of constraints, only one can equal 1. The first set of constraints represent the obligation of having at least *H*^*m *^cells in a row including cell (*i, j*), and only one of them can apply (since only one of variables  equals 1).

#### Vertical constraints

These constraints are built like those for horizontal constraints, exchanging the roles of rows and columns and their respective

### Maximum and minimum height constraints

If a cell is located so that *i *· Δ_*y *_>, it is higher than , and since material *m *should not be located higher than that value, *x*_*ijm *_is to be 0. Minimum height constraints work similarly.

### Accessibility constraints

Materials should be placed within the health workers' reach when needed:(7)

This last set of contraints impose the following conditions. The first term represents the total amount of cells containing material *m *within reach of worker *s*. A cell is out of reach if it is further to the left or to the right than certain values (namely,  and , respectively), regardless of the row where it is located (the sum is for all *i*). If worker *s *needs material *p *in any intervention, the second term of the inequality equals 1, otherwise it equals 0. Therefore, if material is needed (in any intervention) by worker *s*, there should be some cell with that material within his or her reach.

## Results

Running the model in a 1.8 GHz and 2 Gb RAM PC under Windows XP, in order to obtain the optimal solucion to the model, a general solver was used, using Branch and Bound dado que existen muchas variables binarias.

The solver preprocess the model so that redundant constraints are eliminated. The linear relaxation of the model is solved (which means that integrality constraints are overriden). The optimal solution for the linear relaxation typically does not coincide with that of the original model. From that first solution, new constraints are added forcing variables either to be 0 or 1, all variables are binary. This way different subproblems are generated and solved with smaller feasibility regions, untill a binary solucion is obtaind which can proved to be better than any other integer solution.

The values for the parameters of the model where set in accordance to the information withdrawn from the previous stages.

**Figure 3:**
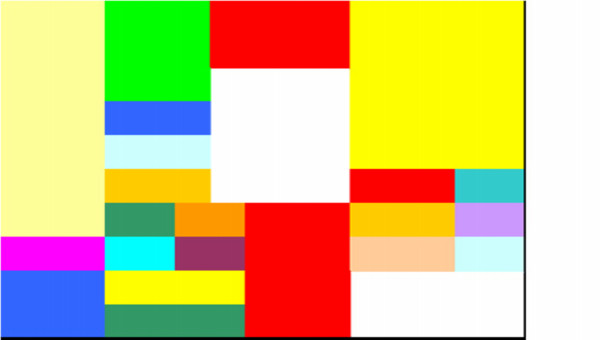
**Grid obtained from the model**. Figure 3 shows the grid obtained after running the linear model from which the eventual layout was obtained

It took 194 minutes to obtained the optimum solution (slightly over 3 hours). As the time to solve the model was more than enough to address the problem dealt with in practical terms, no further alterations of the model where considered so that the computational time might be shorter. The optimal solution can be observed in figure 3, where the location of all different materials have been depicted. No numerical values can be given for extension reasons.

After the optimal solution of the MIP model, the result was sketched in a 3-D program (CATIA) to facilitate the understanding of the whole design, and to make some geometrical analysis with the dummies extending their hands, as the health workers would do in reality (ver Figura 2).

**Figure 4:**
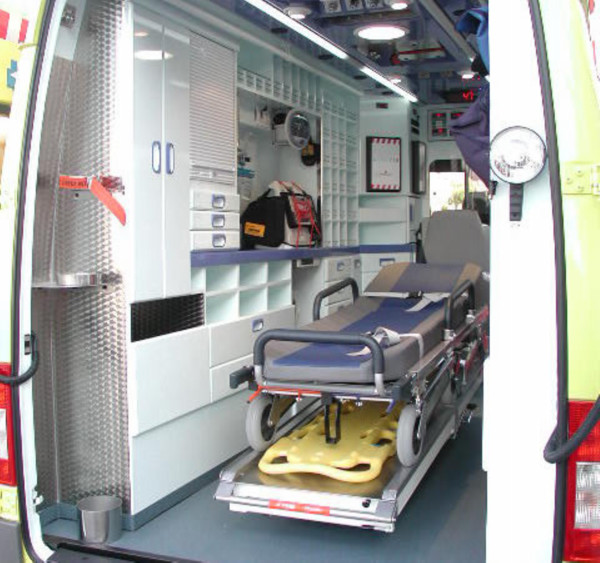
**Rear view of the implemented solution**. Figure 4 shows a rear view of one of the ambulances currently in operation designed according to the results of the study.

**Figure 5:**
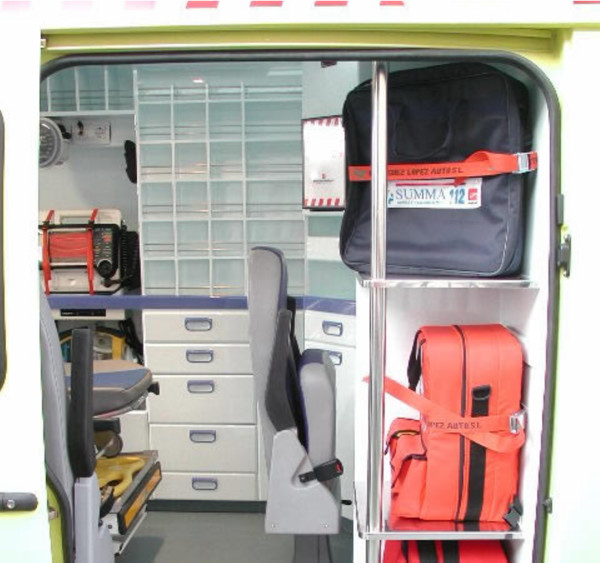
**Lateral view of the implemented solution**. Figure 4 shows a lateral view of one of the ambulances currently in operation designed according to the results of the study.

After discussing this preliminary solution with different staff from SUMMA, a final solution was considered to be the eventual one. Figures 4 and 5 show the implemented solution.

This final layout served to draw up the requirements for the public tender. The body builder who was eventually chosen, provided the first vehicle three months later, so that the health workers could make any suggestions regarding the whole outcome and, in particular, assess the layout obtained as described in this paper. Once all their comments had been gathered, analysed and accepted (if that was the case), the rest of the 22 units were delivered throughout the following months.

After a period of time using the ambulances, most of the health workers consider the results to be highly satisfactory for their needs and requirements, more than any other design they have ever used. However, the coaching team has kept collecting new requests which need to be implemented into the model, so that the next ambulances will meet personnel needs to an even higher degree.

As all the work was done under the coaching group's advice and supervision, it could not be finished until they were satisfied and the result suited their requirements. Moreover, they were of the opinion that the outcome was the best solution they had ever considered.

## Discussion

The model devised to solve the layout problem has proved to be adequate. It was sufficiently accurate to let the researchers obtain a solution. The reasons for the success are mainly the two following ones. Firstly, introducing valid and relevant data into the model was a necessary requirement for the model to offer equally valid output data.

Secondly, the model was built with a level of complexity that would accurately represent the situation under study, but not in such a detailed way that it might become computatonally intractable.

Finally, tuning the solution was done by taking into account some difficult-to-model aspects and some qualitative issues collected throughout the whole project. For instance, a particular material should be located in two different locations. According to the health workers, it would be preferable to have it located in three different locations, each of them very close to each health worker. This was considered when obtaining the eventual layout.

The result has been succesful, as proved by the fact that a public bid made use of the results of this project in order to draw up the specifications for the layout of the patient care space of the ICUs.

## Conclusion

This paper has presented the methodology that allowed the research to obtain a solution for the interior design of the SUMMA 112 ambulance.

A two-step methodology was adopted. Firstly, a multidisciplinary group was assigned the task of identifying the main design criteria and the general outlines of the design.

This first step served as an input for the second phase, which consisted of the development of an optimization model. From this, a first solution was obtained. With some minor alterations to that solution a final feasible proposal was able to be implemented. This final design was included among the requirements of a public tender.

It has been shown that OR can be very useful for that task, making interventions in the ambulance easier and, thus, with a greater probability of success. The same methodology may be equally suitable for other vehicles (helicopters, for example). And furthermore, this approach can be adopted to design other facilities such as operation rooms or any other facility were room is scarce, materials need to be stored and their availability is critical. It is only necessary to analyze the different requirements, namely, the typology of the interventions, the plain walls or sides to allocate the devices, the amount of materials to be carried simultaneously, the position of the health workers, etc, applying the same approach that has been presented. These new conditions involve new parameters and, therefore, a different applied solution.

## Competing interests

The authors declare that they do not have any financial or non-financial competing interest (economic, political, personal, academic, intellectual, commercial or any other) with any organization or person mentioned or involved in the paper.

## Authors' contributions

JSA conducted the data gathering process and the final discussions with the managers to obtain the eventual solution. MOM developed different models to represent the requirements established as a result of the data gathering phase. ÁlGS built and ran the model and interpreted the results. Finally, MGM adapted the solution to obtain the eventual layout for the vehicle.ta All authors have read and approved the final version of the manuscript.

## Pre-publication history

The pre-publication history for this paper can be accessed here:

http://www.biomedcentral.com/1472-6963/9/224/prepub
